# Nintedanib ameliorates animal model of dermatitis

**DOI:** 10.1038/s41598-020-61424-1

**Published:** 2020-03-11

**Authors:** Min-Jeong Heo, Chanmi Lee, Soo Young Choi, Yeong Min Choi, In-sook An, Seunghee Bae, Sungkwan An, Jin Hyuk Jung

**Affiliations:** 1 Korea Institute of Dermatological Science, GeneCellPharm Corporation, 375 Munjeong 2(i)-dong, Songpa-gu, Seoul, 05836 South Korea; 2 Research Institute for Molecular-Targeted Drugs, Department of Cosmetics Engineering, Konkuk University, Seoul, 05029 South Korea

**Keywords:** Target identification, Acute inflammation

## Abstract

Nintedanib, a receptor tyrosine kinase (RTK) inhibitor has been developed as therapeutics for idiopathic pulmonary fibrosis and non-small lung cancer. We found that the expression levels of RTK, especially VEGFR1 is increased in skin biopsies of dermatitis patients from multiple independent datasets. Moreover, VEGFR1 is highly expressed by infiltrated cells in dermis from oxazolone (OXA) treated mice. Interestingly, nintedanib alleviates dermatitis symptom in OXA-induced animal model. Especially, levels of epidermis thickness, infiltrated immune cells including mast cells and eosinophils were decreased from mice cotreated with nintedanib and OXA compared with OXA treated mice. Moreover, serum IgE and Th2 cytokines including IL-4 and IL-13 were decreased by nintedanib treatment. These results suggest an evidence that nintedanib alleviates animal model of dermatitis.

## Introduction

**Figure 1:**
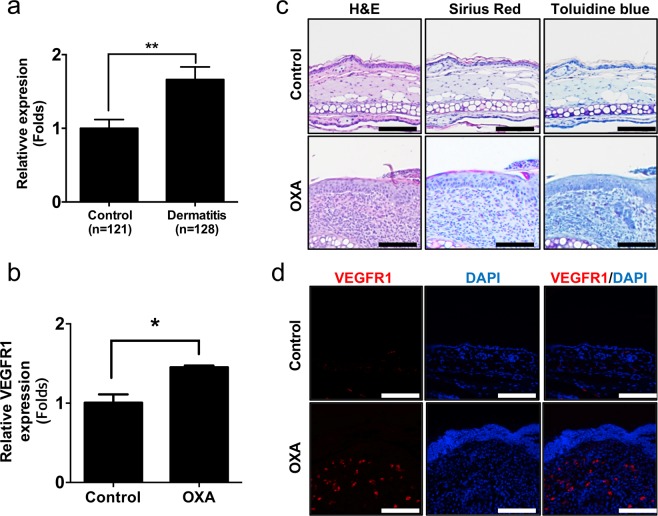
The expression levels of VEGFR1/*FLT1* in animal model of dermatitis and dermatitis patients. (**a**) Relative expression levels of *FLT1* in normal (n = 121) and dermatitis patients (n = 128). (**b**) mRNA expression of *Flt1* was measured in oxazolone (OXA) treated mice ear and vehicle control (n = 15). (**c**) Histological analysis (H&E, Sirius red and toluidine blue staining) of control and oxazolone treated mice ear. Scale bar = 100 μm. (**d**) Immunofluorescent analysis of VEGFR1 from control and oxazolone treated mice ear. Scale bar = 100 μm. Data are presented as mean ± SEM and analyzed by student t-test. *p  <  0.05, **p  <  0.01 compared to control.

Receptor tyrosine kinases (RTKs) are membrane bound receptors for growth factors and hormones that modulate cellular process to have a crucial role in the development^1,2^. Because RTKs also often overexpressed in cancers including breast and non-small lung cancers, many inhibitors against RTKs have been developed for anticancer treatments^3^. On the other hands, tyrosine kinase mediates the signal from various immune related receptors including leukocyte antigen receptors, innate immune receptors, and cytokine receptors to activate immune cells and recruit to inflammation lesion^4^. Autoimmune and inflammatory diseases are characterized by inflammatory microenvironment and tyrosine kinase serves essential role in immune-mediated disorders^5^. Therefore, small molecules targeting tyrosine kinase have been developed for autoimmune and inflammatory diseases^6–8^. Especially JAK is a one of primary tyrosine kinases for therapeutic target since JAK is responsible for numerous cytokines expression via type I/II cytokine receptor signaling^9–11^. Many JAK inhibitors are FDA approved in clinic use for autoimmune and inflammatory diseases^12^. Atopic dermatitis (AD) is one of the most common skin inflammatory diseases affecting 3–10% adults and 15–20% of children in USA^13–15^. AD pathogenesis is a complex of skin barrier dysfunction, alteration of immune responses, IgE-mediated hypersensitivity^16,17^. Treatments of atopic dermatitis are non-specific immunosuppressants and Th2 specific therapies including biologics^18–20^. Because IL-4, which is a primary pathogenic in AD requires JAK1 and 3 with additional complex, JAK inhibitors including tofacitinib and baricitinib have been determined their efficacy on the AD^21–23^. VEGFR1 is a receptor of VEGF, transduces a signal to induce angiogenesis and lymphangiogenesis^24,25^. Dilated vessels and perivascular edema are frequently found in AD lesion with erythema^26,27^. Moreover, increased levels of VEGF are found in plasma and AD lesion^28,29^. Although VEGF-VEGFR signaling is highly activated in AD, use of VEGFR inhibitor for AD treatment remains unexplored. Interestingly, we found expression levels of RTKs are upregulated in skin biopsies of dermatitis patients from several independent datasets (Fig. 1 and Table S1). Nintedanib is a potent receptor kinase inhibitor that competitively binds to the kinase domains of vascular endothelial growth factor receptor (VEGFR), platelet-derived growth factor receptor beta (PDGFRB) and fibroblast growth factor receptor (FGFR)^30^. Nintedanib is a FDA-approved oral drug for idiopathic pulmonary fibrosis (IPF)^31^. Here we used nintedanib to determine whether nintedanib attenuates oxazolone-induced animal model of dermatitis.

## Material and Methods

All methods were performed in accordance with the relevant guidelines and regulations.

### Experimental animals

7-week-old BALB/c mice were purchased from the Central Laboratory Animals (Seoul, Korea). They were feed and raised under specific pathogen-free conditions with 12 h light/dark cycle in controlled environment condition as temperature and humidity. All experimental procedures were approved by the Institutional Animal Care and Use Committee of the Konkuk University (KU19160).

### OXA-induced murine model of dermatitis

**Figure 2:**
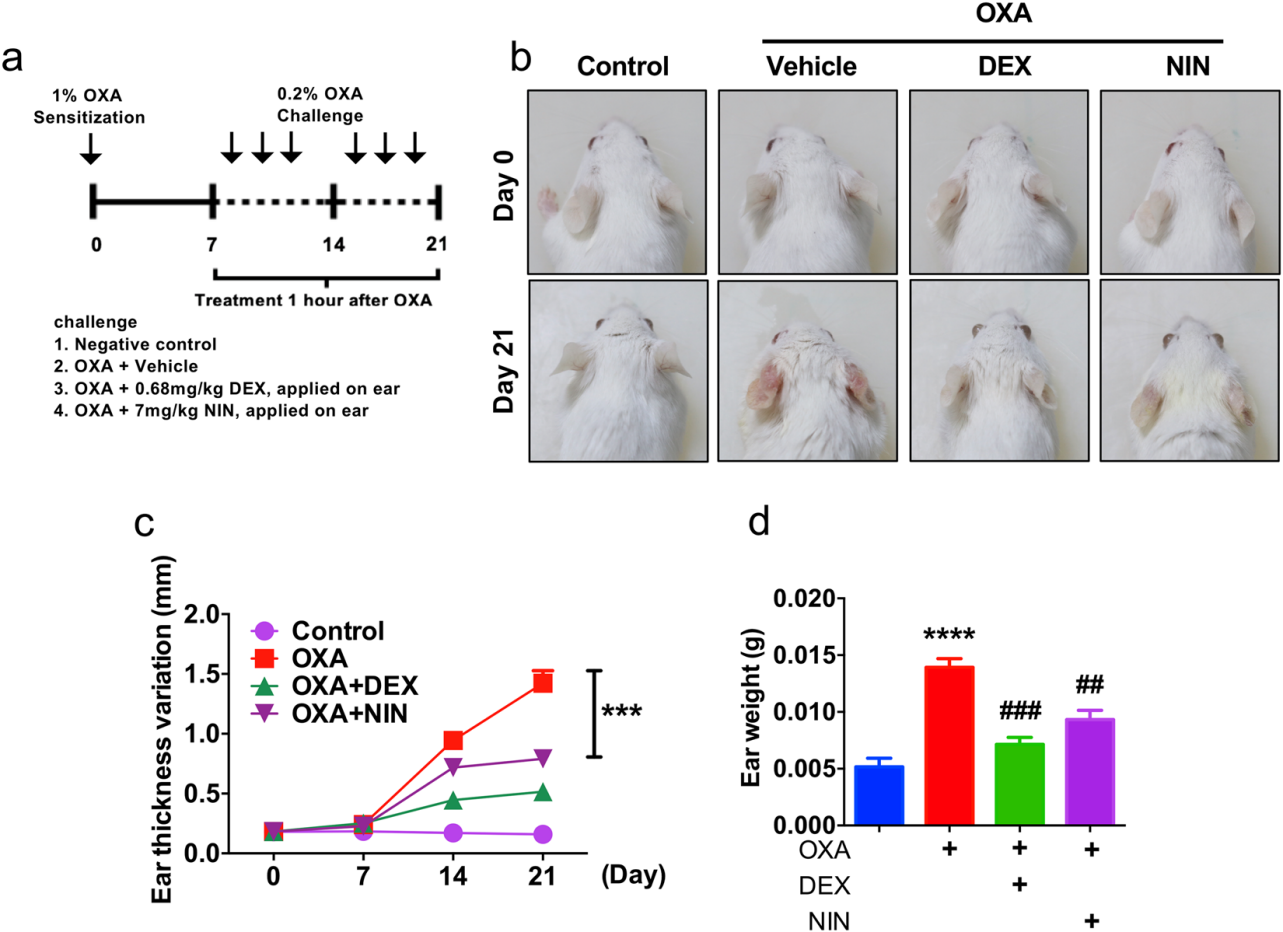
Morphological analysis of nintedanib-treated mice. (**a**) Schematic diagram of oxazolone (OXA)-induced animal model. Four groups: untreated controls, OXA only and mice treated with DEX (Dexamethasone) or nintedanib (NIN) one hour after every OXA challenge. (**b**) Representative photographs of mouse ears from each group on day 0 and 21. (**c**) Ear thickness was measured every week as indicated. Data are from three independent experiments (n = 15). Data are presented as mean  ±  SEM. Data (Day 21) are analyzed by student t-test. ***p  <  0.005 compared to oxazolone treated. (**d**) Ear weight was measured at day 21. Data are from three independent experiments (n = 15). Data are presented as mean  ±  SEM and analyzed by one-way ANOVA (****p < 0.001 compared to control, ^##^p < 0.01, ^###^p < 0.005 compared to oxazolone treated).

To induce AD-like lesion, all ears of mice except the negative control group were applied with oxazolone (OXA). OXA-induced animal model of dermatitis was performed according to previously described with some modifications^32,33^. Briefly, skin inflammation was induced by topical administration with 30 μl of 1% OXA dissolved in acetone (Merck, Kenilworth, USA) on day 0, and repeated 30 μl of 0.2% OXA three times a week from day 7 to 21. Negative control group was treated same volume of vehicles. Therapeutic groups as 0.68 mg/kg dexamethasone (DEX) or 7 mg/kg Nintedanib (NIN) were applied on ear after 1 h every challenge. Drug administration was performed under light anesthesia with isoflurane. Detailed description is as shown Fig. 2a. Mice were photographed by Digital Single-Lens Reflex camera (Conditions; F5.6 1/40, ISO800; Canon, Tokyo, Japan).

### Ear thickness and ear weight

Ear thickness was measured every other day using digital micrometer (Japan), and ear was collected using 5-mm biopsy punch (KAI Medical, Japan) after sacrifice for measuring weight on day 21.

### Histology

Ear tissues were fixed in 10% formalin solution (Sigma, Mo, USA) for 24 h. Tissues were washed with PBS (Biosesang, Seongnam, Korea), embedded in paraffin (Leica Biosystems, Wetzlar, Germany) and sectioned 4 μm thickness using microtome (HistoCore BIOCUT; Leica Biosystems, Germany). Stained images were taken using light microscope (Olympus, Tokyo, Japan). Mast cells or eosinophils were counted in 3 random high-power field (HPF) each mouse at 400× magnification. H&E staining was performed according to previously described^34^. Epidermis and dermis thickness were measured using imageJ software program. Toluidine blue staining was used to count infiltrated mast cells. Briefly, hydrated tissue sections were stained with 0.1% Toluidine Blue O in 1% sodium chloride solution (pH 2; Sigma, USA) for 1 min. After staining, sections were washed briefly in PBS, and dehydration 3 times with 95% to 100% ethanol, and then sealed using mounting medium^35^. Sirius Red staining was used to count the number of infiltrated eosinophil cells^36^. Reagent preparation and staining protocol were according to previously as described^37^.

### Cells and reagents

NIH3T3/NKκB-luc cell line was purchased from Panomics (RC0015). Cells were maintained in a humidified incubator at 37 °C and 5% CO_2_. FBM^TM^-2 Fibroblast Growth Medium-2 BulletKit^TM^ was purchased from Lonza (CC-3132, Lonza). Recombinant Human TNF-alpha was purchased from Peprotech (300-01A-10, Peprotech). Bay was purchased from Sigma (11-7082, Sigma). Nintedanib was purchased from Cayman chemical (11022).

### Cell viability assay

Viability was performed as previously described with slight modification^38^. Briefly, 1 × 10^4^ cells were plated in a 96 well plate. Pre-incubate the plate for 24 h in a humidified incubator. After nintedanib treatment, cells were incubated with mixture (1:10) of EZ-Cytox cell viability assay kit (Dogen, EZ3000). Then plate was incubated for 30 min in the incubator and determined absorbance at 450 nm with reference to 655 nm wavelength (iMark, Biorad).

### Luciferase assay

Luciferase assay was performed as previously described with slight modification^39^. 6 × 10^5^ cells were seeded in 24-well plates treated 50 nM TNF-α for 8 h with or without nintedanib. Cells were harvested and cell extracts were prepared using 100 μl of passive lysis buffer (Promega). Luciferase activities were measured using Veritas Luminometer (Turnur Designs, Sunnyvale, CA, USA).

### Web-based meta-analysis

Microarray datasets from studies (GSE60028^40^, GSE79150^41^, GSE36842^42^, GSE46239, GSE32924^43^, GSE121212^44^, GSE16161^45^, GSE120721^46^ and GSE107361^47^ were analyzed using GEO2R (https://www.ncbi.nlm.nih.gov/geo/geo2r) to determine enzyme expression of RTK.

### Serum IgE ELISA

Serum was collected from the abdominal aorta of mice. Whole blood was placed during 30 min at room temperature and centrifuged at 4 °C for 15 min at ×12,000 rpm. The supernatant was stored immediately at −80 °C. For analysis, samples were diluted to 1/200 with assay diluent. Total IgE were determined by ELISA kit according to manufacturer’s manual (BD Pharmingen; San Diego, CA, USA). All measurements were analyzed by optical density at 450 nm.

### RNA isolation and RT-PCR

Mice ear was homogenized in 1 ml of TRIzol reagent (Takara Co., Ltd. Japan) using IKA-T10 basic homogenizer (ULTRA-TURRAX; 10 mm; IKA Laboratory Equipment, Staufen, Germany). Total RNA was isolated previously described^48^. Total RNA was quantified by MaestroNano Micro-Volume Spectrophotometer (Xiangshan Diist, Taiwan) and 300 ng RNA was used for reverse transcription using M-MLV reverse transcriptase (Invitrogen, California, USA). Real-time PCR analysis was performed with duplicate using SYBR® Master Mix in the BIOER Real-Time PCR machine (Fluorescent Quantitative Detection systems; Hangzhou, China). For calculation efficiency of the amplification, the relative quantitative of each target gene was normalized to the housekeeping gene as β-Actin. Data was calculated by the 2^− ΔΔCT^ method based on the normalization gene of control group.

### Immunofluorescence

Immunofluorescence was performed using a heat-induced epitope retrieval (HIER) protocol with slight modification^49^. After deparaffinization, slides were placed in a plastic container filled with citrate buffer, pH 6.0 at 60 °C for 20 min. And then, slides were allowed to cool for 20 min at room temperature and were then rinsed with phosphate-buffered saline with Tween 20 (PBST, Duchefa Biochemie, Hofmanweg, Netherlands). Slides were incubated in blocking buffer (BLOXALL® Endogenous Peroxidase and Alkaline Phosphatase Blocking Solution, Vector Laboratories, Inc., California, USA) for 1 h at room temperature to remove non-specific binding. Next, they were incubated for 24 h with Flt1 (VEGFR1) antibody (Santacruz biotechnology, Texas, USA) in blocking buffer at 4 °C. Next day, the slides were washed and incubated for 1 h at room temperature with anti-mouse secondary antibodies conjugated with Alexa Flour 647 (Abcam, Cambridge, United Kingdom). The slides were counterstained with DAPI for 10 min and mounted with fluorescence mounting medium (Agilent, California, USA).

### Statistical analysis

All statistical evaluations were performed using Prism 6 (GraphPad Software, La Jolla, CA). Data are given as mean ± standard error of the mean (SEM). Statistical significance was analyzed using Student’s *t*-test and one-way ANOVA. *P* values of <0.05, <0.01 and <0.001 were considered as statistically significant differences.

### Ethics approval and consent to participate

All animals were care for by using protocols approved by the Institutional Animal Care and Use Committee (Konkuk University, Republic of Korea). No. KU10160.

## Results

### Vascular endothelial growth factor receptor1 (VEGFR1) is highly expressed in atopic dermatitis lesion

We determined the levels of RTKs (VEGFR1, PDGFRB and FGFR2) from dermatitis patients and normal controls using meta-analyses. Interestingly, the expression levels of VEGFR1 were increased from ten studies from seven independent datasets of lesion from dermatitis patients compared with control subjects (Fig. 1a and. Table S1) (GSE60028^40^, GSE79150^41^, GSE36842^42^, GSE46239, GSE32924^43^, GSE121212^44^, GSE16161^45^). We found PDGFRB and FGFR2 were increased from dermatitis patients compared to normal control (Table S1). Moreover, we found the levels of VEGFR1 are increased in oxazolone (OXA) treated mice (Fig. 1b). Consequently, VEGFR1 was increased by infiltrated cells in dermis of OXA treated mice (Fig. 1c,d). These results indicated that expression of RTKs, especially VEGFR1 is increased in dermis of atopic dermatitis lesion.

### Nintedanib ameliorates dermatitis in OXA-induced animal model

To determine whether nintedanib, RTK inhibitor is effective on dermatitis, we employed OXA-induced mice model (Fig. 2a). We found nintedanib treatment is able to attenuate morphological phenotype including skin redness and swelling of OXA-induced skin inflammation (Fig. 2b). Moreover, the levels of ear thickness and weight robustly decreased from mice cotreated with nintedanib and OXA compared to OXA-treated mice (Fig. 2c,d).

### Nintedanib attenuates OXA-induced dermatitis in histological analysis

**Figure 3:**
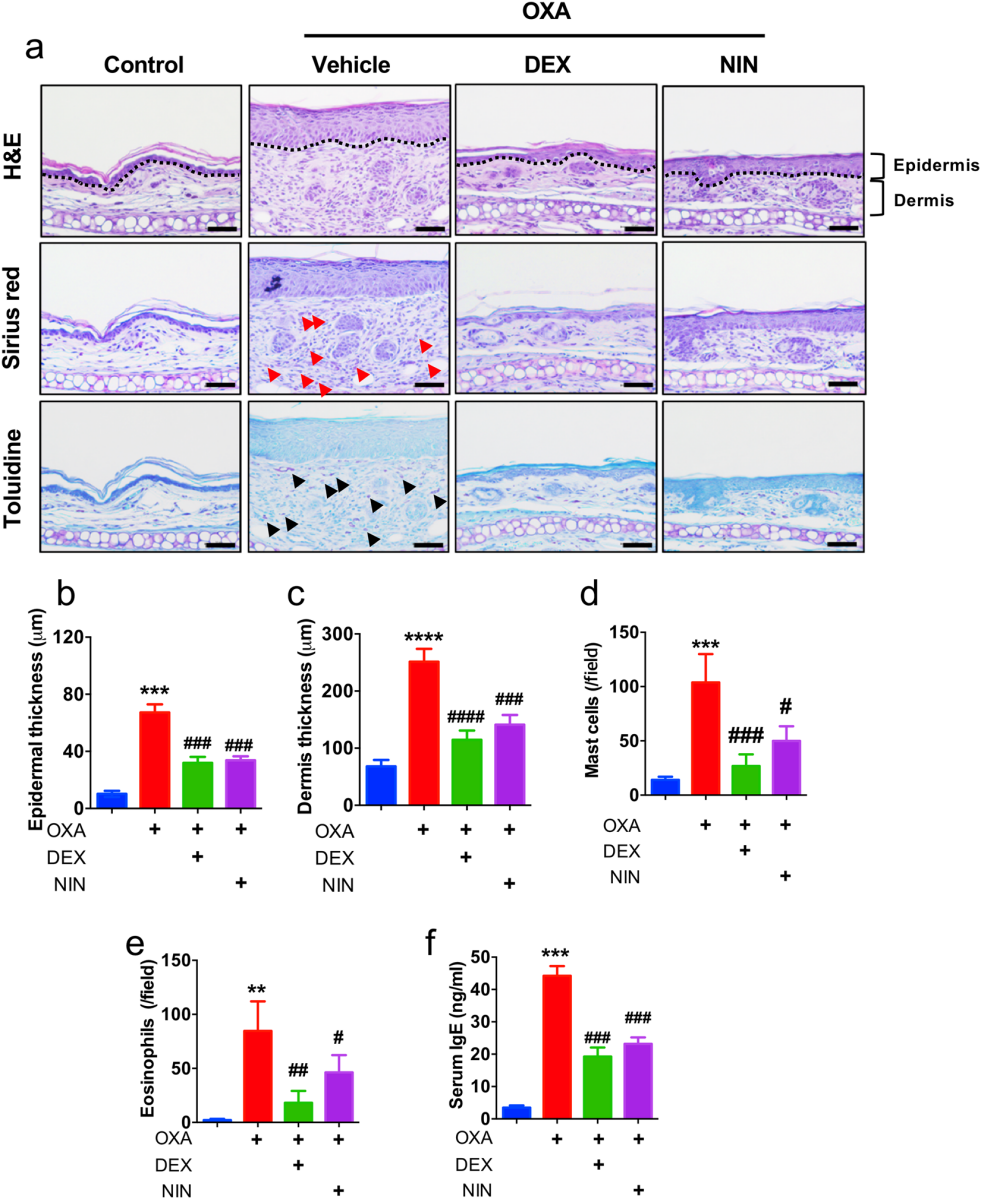
Histological analysis of nintedanib-treated mice. **(a**) H&E, sirius red and toluidine blue staining in ear lesions. Scale bar = 50 μm (**b**) Mean of epidermal thickness was measured using three different sections. (**c**) Mean of epidermal thickness was measured using three different sections. (**d**) Mean of mast cells (black arrow in toluidine blue staining) in dermis was measured. (**e**) Mean of eosinophil cells (red arrow in sirius red staining) in dermis was measured. (**f**) Serum IgE level was measured by ELISA at day 21. Data are from three independent experiments (n = 15). Data are presented as mean  ±  SEM of changes in values and analyzed by one-way ANOVA (**p < 0.01, ***p < 0.005, ****p < 0.001 compared to control, ^#^*p* < 0.05, ^##^p < 0.01, ^###^p < 0.005, ^####^*p* < 0.001 compared to oxazolone treated).

We found epidermis and dermis thickness are decreased from mice cotreated with nintedanib and OXA using histological analysis (Fig. 3a,b). Interestingly, numbers of infiltrated mast cells as well as eosinophils into dermis were decreased from mice cotreated with nintedanib and OXA compared to OXA-treated (Fig. 3d,e). Moreover, serum IgE levels were decreased from mice cotreated with nintedanib and OXA compared to OXA-treated mice (Fig. 3f). These results indicated that topical administration of nintedanib ameliorates OXA-induced dermatitis by histological analysis.

### Nintedanib attenuates cytokine expression in OXA-induced model of dermatitis

**Figure 4:**
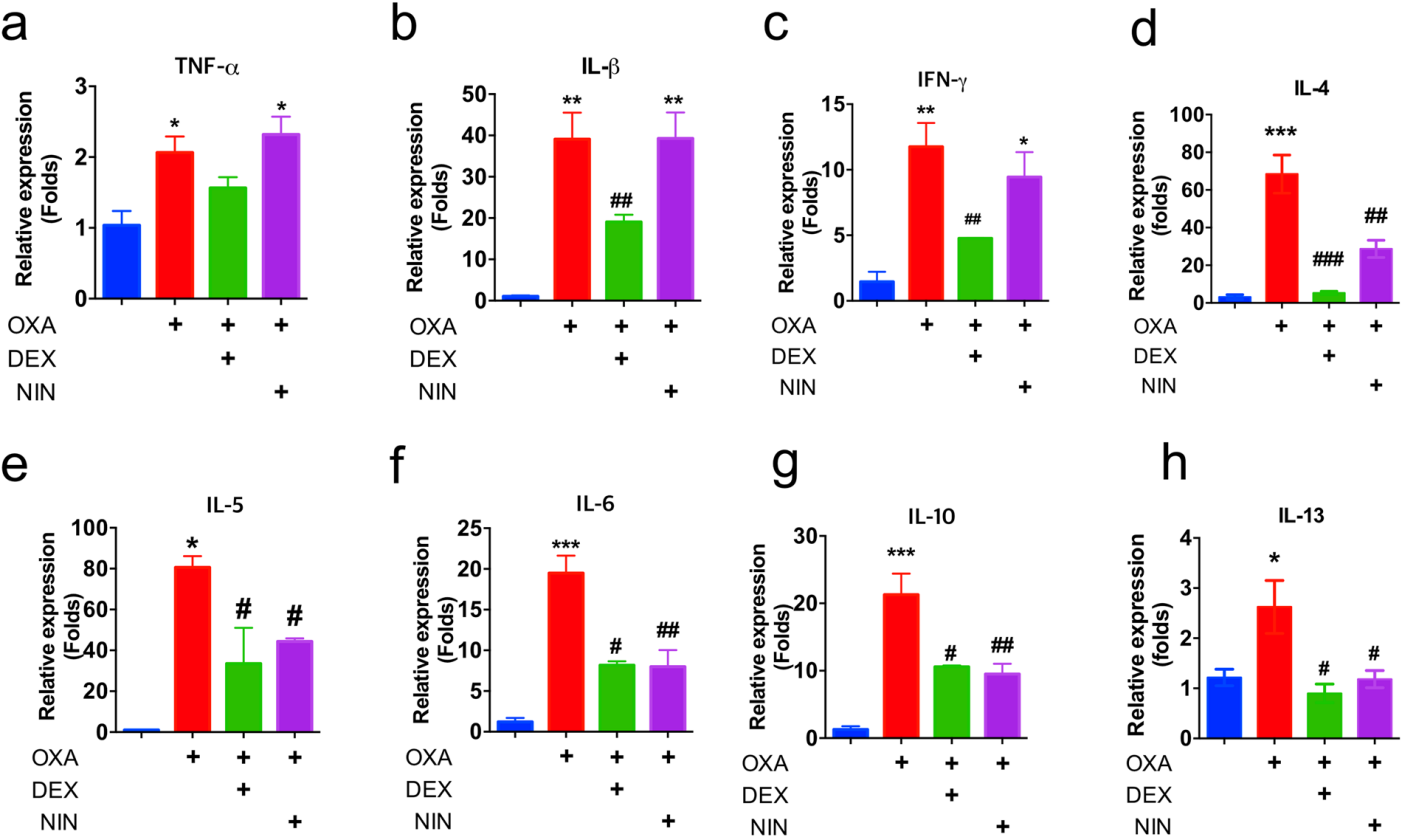
Expression of cytokines in nintedanib-treated mice. (**a)** mRNA levels of TNF-α from indicated mice ear. (**b)** mRNA levels of IL-β from indicated mice ear. (**c**) mRNA levels of IFN-γ from indicated mice ear. (**d**) mRNA levels of IL-4 from indicated mice ear. (**e**) mRNA levels of IL-5 from indicated mice ear. (**f**) mRNA levels of IL-6 from indicated mice ear. (**g**) mRNA levels of IL-10 from indicated mice ear. (**h**) mRNA levels of IL-13 from indicated mice ear. Data are from three independent experiments (n = 15). Data are presented as mean  ±  SEM and analyzed by one-way ANOVA (*p < 0.05, **p < 0.01, ***p < 0.005 versus control) and (^#^*p* < 0.05, ^##^p < 0.01 versus OXA).

Th2-type cytokines including IL-4 and IL-13 are one of the typical markers as well as therapeutic targets of atopic dermatitis^20^. We therefore, analyzed expression of cytokines from mice ear to determine whether nintedanib attenuates immune response. Interestingly, Th2 cytokines including IL-4, IL-5, IL-6, and IL-13 were decreased from mice cotreated nintedanib with OXA while there was no change on the expression of Th1 cytokines including TNF-α, IL-1β and IFN-γ (Fig. 4a–h). These results indicated that nintedanib attenuates Th2-type immune response in oxazolone-induced animal model of dermatitis. In order to determine the molecular mechanism of nintedanib-mediated anti-inflammatory effect, we used 3T3 murine fibroblasts, which stably expressed luciferase reporter plasmid encoded with NFκB-binding motif. Because 100 nM nintedanib-treated fibroblasts showed 91% viability, 50 nM and 100 nM nintedanib were used to measure NFκB activity in presence of TNF-α (Fig. S2a). We found that nintedanib is not able to modulate NFκB activity (Fig. S2b). These results indicated that NFκB may not be the primary molecular signaling pathway for nintedanib to inhibit oxazolone-induced animal model of dermatitis.

## Discussion

We found the expression levels of RTKs especially, VEGFR are increased in the lesion of dermatitis patients (Fig. 1a and Table S1). There are numerous reports that VEGFR is highly expressed during inflammation and further studies are required to determine the role of VEGFR in lesion of dermatitis patients^50–52^. Moreover, systemic anti-VEGF treatments strongly reduced skin inflammation in a mice model of psoriasis^53^. Those observations drove us to use nintedanib a potent FDA approved RTK inhibitor to determine therapeutic efficacy in animal model of dermatitis. We found that nintedanib treatment attenuates phenotype of oxazolone-induced skin inflammation in animal model without toxicity (Figs. 2b and S1). Histological analysis showed that nintedanib reduces infiltrated immune cells including mast cells and eosinophils (Fig. 3d,e). Moreover, the levels of Th2-type cytokines expression including IL-4 and IL-13 were reduced from nintedanib with OXA treated mice ear compared to OXA-treated (Fig. 4d-h). NFκB signaling is an important signaling pathway that orchestrates inflammatory response^54^. We found that nintedanib is not able to attenuate NFκB activity in presence of TNF-α (Fig. S2a,b). According to recent reports, nintedanib inhibits angiogenesis, reduced M (IL-4) cell polarization and induces apoptosis of mesenchymal cells during fibrotic remodeling^55–58^. Thus, we speculated that nintedanib may regulate migration of inflammatory cells into the AD lesion by inhibiting angiogenesis, and/or immune cells polarization. Adverse effects of topical use of steroid-containing cream have been well established^59,60^. Although, novel drugs have been developed for dermatitis therapeutics including PDE4 inhibitors and JAK inhibitors, high costs of new drugs are one of the major burdens while dermatitis patients are increasing every year^61,62^. Therefore, repurposing drugs may cover these limitations of atopic dermatitis therapeutics. And nintedanib could be a one of the primary candidates as a repurposing drug because effects of nintedanib on air way inflammation is reported beyond current indication^63^. Future studies are remains to be explored to determine molecular mechanism of nintedanib-mediated anti-inflammatory effect on dermatitis.

## Supplementary information

Supplementary information.

## Data Availability

All study data are available from the corresponding author upon request.
