# Supplementary material for: Nintedanib ameliorates animal model of dermatitis
Source: Sci Rep. 2020 Mar 11;10:4493. doi: 10.1038/s41598-020-61424-1 (PMC7066145; doi:10.1038/s41598-020-61424-1)
Supplement: Supplementary file 1 — Supplementary information. [file 41598_2020_61424_MOESM1_ESM.pdf]

SUPPLEMENTARY INFORMATION

Nintedanib ameliorates animal model of dermatitis

Min-Jeong Heo[1], Chanmi Lee[1], Soo Young Choi[1], Yeong Min Choi[1], In-sook An[1], Seunghee Bae[2], Sungkwan An[2*], Jin Hyuk Jung[1*]

[1]Korea Institute of Dermatological Science, GeneCellPharm Corporation, 375 Munjeong 2(i)-dong, Songpa-gu Seoul 05836, South Korea. [2]Research Institute for Molecular-Targeted Drugs, Department of Cosmetics Engineering, Konkuk University, Seoul 05029, South Korea.

*Corresponding authors

Corresponding Authors details

Jin Hyuk Jung Ph.D. Principal investigator, Korea Institute of Dermatological Science, GeneCellPharm. +82-2-70-7797-2927 (Tel), jungjh@skinreseach.or.kr

Sungkwan An Ph.D. Professor, Department of Cosmetics Engineering, Konkuk University. +82-2-450-4054 (Tel), ansungkwan@konkuk.ac.kr

List of Supplementary Materials

Supplementary Table 1, related to Figure 1

Supplementary figure 1, related to Figure 2

Supplementary figure 2, related to Figure 4

Supplementary Table 2, related to Methods

Table S1. Meta-analysis of RTK expression in skin biopsies from dermatitis patients and control subjects

Table S1. Meta-analysis of RTK expression in skin biopsies from dermatitis patients and control subjects

Gene	Imported Id	Rank	Fold Change	P-Value	Test Expression	Control Expression	Samples	GSE number	PMID
FLT1	226498_at	102	2.9	0.0333	1234.3	425	Acute lesional skin of atopic dermatitis patients - family history of atopy _vs_ no	GSE36842	22951056
FLT1	226497_s_at	951	1.89	0.0432	1258.9	666.3			
FLT1	222033_s_at	1336	1.39	0.0336	4950.9	3566.2	Skins from contact dermatitis patients - cobalt patched skin test _vs_ control (petrolatum)	GSE60028	24768652
FLT1	226498_PM_at	1691	1.83	0.0063	1473.2	803.5	Skin biopsies of hidradenitis suppurativa patients at baseline - lesional skin _vs_ non-lesional	GSE79150	26707687
FLT1	226497_PM_s_at	2380	1.68	0.017	5077.8	3020.9			
FLT1	222033_PM_s_at	2835	1.61	0.0036	4254.8	2641			
FLT1	222033_s_at	4321	2.98	0.0005	3082.8	1034.3	Skin - chronic lesion of atopic dermatitis _vs_ healthy	GSE36842	22951056
FLT1	222033_s_at	4510	3.42	3.00E-06	3536.5	1033.1	Skin - acute lesion of atopic dermatitis _vs_ healthy		
FLT1	222033_s_at	4584	1.33	0.0449	1169.1	881.9	Skin of dermatomyositis patients _vs_ healthy controls	GSE46239	
FLT1	222033_s_at	5118	1.33	0.0416	1540.3	1158.9	Skin biopsy nonlesional of patients with	GSE32924	21388663
FLT1	2321	2813	1.5287	7.60E-06	1.7026	1.0093	Skin biopsies - lesional of atopic dermatitis patients _vs_ healthy control subjects	GSE121212	30641038
FLT1	222033_s_at	9354	1.29	0.0327	1547.6	1196.5	Skin biopsy from atopic dermatitis lesion _vs_ normal skin	GSE16161	20004782
FLT1	226498_at	9654	1.28	0.0139	1013.5	794.7			
FLT1	2321	5044	1.2478	0.0075	1.6893	1.3634	Skin biopsies of atopic dermatitis patients - lesional _vs_ matched non-lesional	GSE121212	30641038
PDGFRB	202273_at	2537	1.3	0.0425	6425.2	4933.1	Skin lesions (acute and chronic) of moderate-to-severe atopic dermatitis patients	GSE36842	22951056
FGFR2	203639_s_at	5006	1.94	0.0091	925	476.7	Epidermis from lesional skin of atopic dermatitis patients _vs_ normal healthy skin	GSE120721	25567045
FGFR2	203639_s_at	9777	1.28	0.0204	1559.1	1214.8	Skin tissues of pediatric patients with atopic dermatitis - lesional _vs_ non-lesional (matched)	GSE107361	29731129

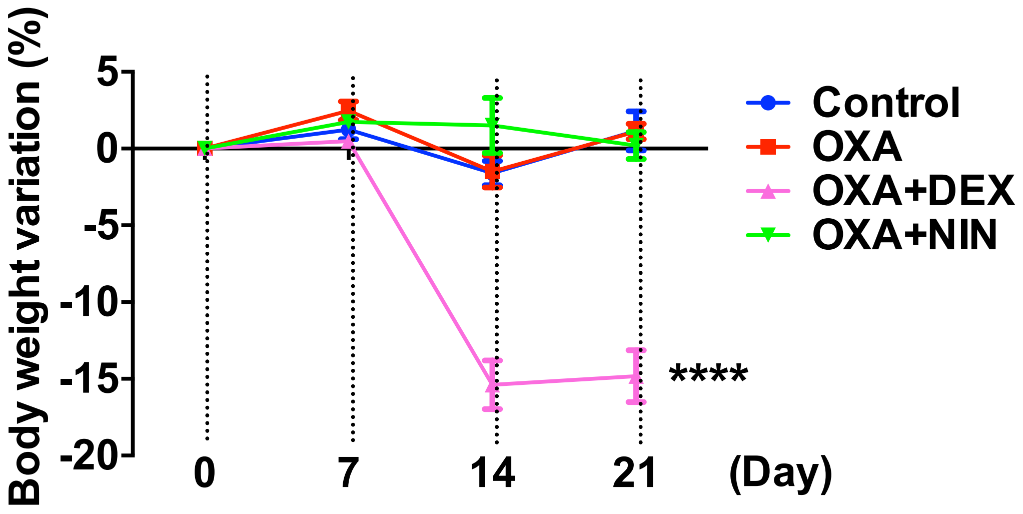

Fig. S1 Body weight variation in oxazolone-induced animal model of dermatitis.

Mice weight was measured at indicated days and percentage of weight change were calculated based on day 0. Data are from three independent experiments (n=15). Data are presented as mean ± SEM. Data (day 21) are analyzed by one-way ANOVA (****p<0.001 compared to control).

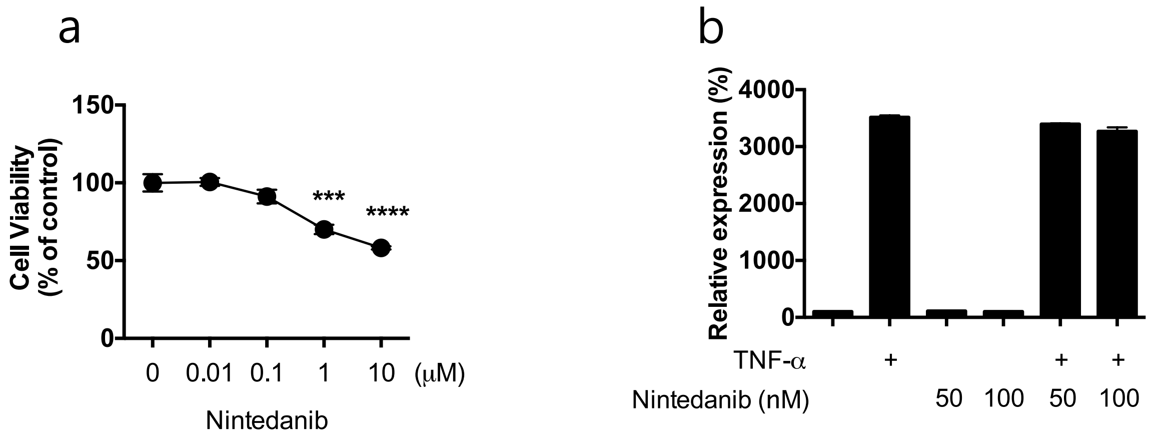

Fig. S2 Nintedanib is not able to modulate NFκb activity *in vitro* (a) Viability of fibroblasts after indicated concentration of nintedanib treatment for 8h. (b) Luciferase assay after 8h of incubation with TNF-α with indicated concentration of nintedanib. Data are from three independent experiments (n=9). Data are presented as mean ± SEM of changes in values and analyzed by one-way ANOVA (***p<0.005, ****p<0.001 compared to control).

Table S2. List of primers used for Quantitative real-time PCR

Gene	Forward (5' to 3')	Reverse primer (5' to 3')
TNF-α	CTACTCCTCAGAGCCCCCAG	TGACCACTCTCCCTTTGCAG
IFN-γ	CCATCGGCTGACCTAGA	GCCACTTGAGTTAAAATAGTTAT TCAGAC
IL-1β	GAAAGACGGCACACCCACCCT	GCTCTGCTTGTGAGGTGCTGATG TA
IL-4	ACAGGAGAAGGGACGCCA	GAAGCCCTACAGACGAGCTCA
IL-5	GGCTGGCCTCAAACTGGTAA	CCCTGATGCAACGAAGAGGA
IL-6	GTGTAATTAAGCCTCCGACTTG	TCCAGTTGCCTTCTTGGGAC
IL-10	ATAACTGCACCCACTTCCCA	GGGCATCACTTCTACCAGGT
IL-13	GCAACGGCAGCATGGTATGGA	TGGTATCGGGGAGGCTGGAGAC
β-Actin	GTATGGAATCCTGTGGCATC	AAGCACTTGCGGTGCACGAT
